# Complex Genomic Rearrangements following Selection in a Glutamine-Limited Medium over Hundreds of Generations

**DOI:** 10.1128/mra.00729-22

**Published:** 2022-10-27

**Authors:** Pieter Spealman, Grace Avecilla, Julia Matthews, Ina Suresh, David Gresham

**Affiliations:** a Center for Genomics and Systems Biology, Department of Biology, New York University, New York, New York, USA; University of Maryland School of Medicine

## Abstract

Large-scale genomic changes, including copy number variations (CNVs), are frequently observed in long-term evolution experiments (LTEEs). We have previously reported the detection of recurrent CNVs in Saccharomyces cerevisiae populations adapting to glutamine-limited conditions over hundreds of generations. Here, we present the whole-genome sequencing (WGS) assemblies of 7 LTEE strains and their ancestor.

## ANNOUNCEMENT

Large-scale genomic rearrangements frequently occur in single-celled organisms adapting to nutrient-limited conditions (1, 2). We previously adapted populations of Saccharomyces cerevisiae to nutrient-limited growth medium over hundreds of generations (3). We present the whole-genome sequencing (WGS) genome assemblies of 7 evolved strains of Saccharomyces cerevisiae and their ancestor.

**FIG 1:**
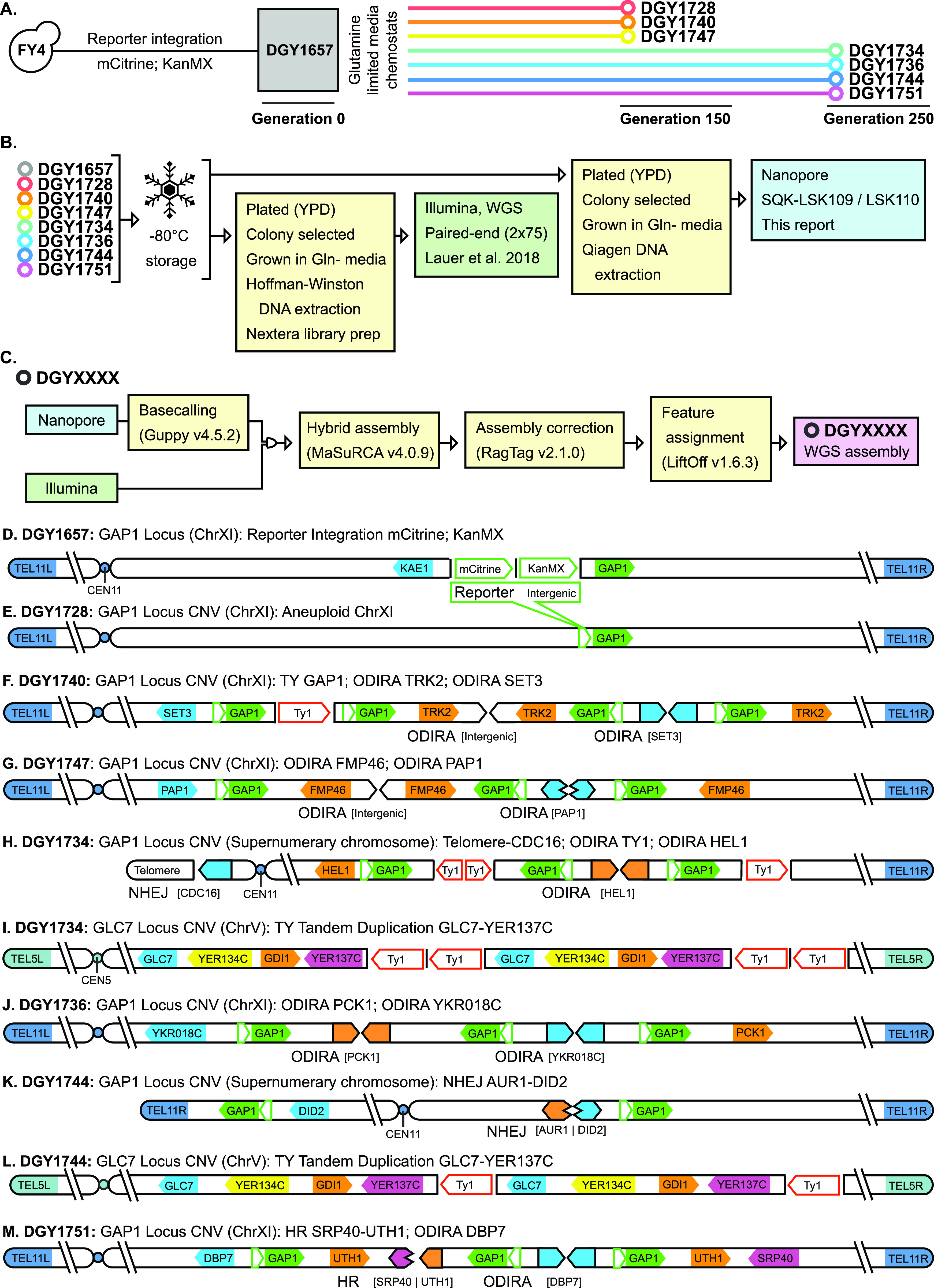
Schematic showing S. cerevisiae strain provenance. (A) Ancestral strain DGY1657 has a copy number variation (CNV) reporter construct in a FY4 background. DGY1657 was inoculated into separate chemostats and grown in glutamine-limited medium. The strains were isolated and sequenced at generations 150 (DGY1728, DGY1740, DGY1747) and 250 (DGY1734, DGY1736, DGY1744, DGY1751). (B) Flowchart showing the strain and independent library preparation paths for Illumina (green box) and Nanopore (blue box). (C) Flowchart for hybrid WGS assembly pipeline for a given strain. (D to M) Topology diagrams for evolved strains indicating CNV breakpoints, orientations, and the occurrence of transposon events. Green boxes represent the reporter; red arrows indicate transposon yeast. CNV breakpoints are annotated with their most likely mechanism. ODIRA, origin-dependent inverted-repeat amplification; NHEJ, nonhomologous end-joining; HR, homologous recombination.

The ancestor strain (DGY1657) is derived from the S288C haploid strain FY4 (4, 5), with a constitutively expressed fluorescent reporter (*ACT1pr*::*mCitrine*::*ADH1term*) and a drug resistance gene (*TEFpr*::*KanMX*::*TEFterm*) integrated into the upstream intergenic region of *GAP1* (chromosome XI [ChrXI], position 513945). We inoculated DGY1657 into 20-mL chemostat vessels (6) containing glutamine-limited (Gln−) medium (400 μM glutamine, 1 g/L CaCl_2_·2H_2_O, 1 g/L NaCl, 5 g/L MgSO_4_·7H_2_O, 10 g/L KH_2_PO_4_; 2% glucose, metals, vitamins [7]). Chemostats were maintained at 30°C in aerobic conditions and diluted at a rate of 0.12 h^−1^ (approximate doubling time, 5.8 h). Steady-state populations of 3 × 10^8^ cells were maintained and sampled at generations 150 (~870 h; DGY1728, DGY1740, DGY1747) and 250 (~1,450 h; DGY1734, DGY1736, DGY1744, DGY1751) (Fig. 1A). Clones from these populations were isolated by plating the cultures onto rich medium (yeast extract-peptone-dextrose [YPD]; 30°C, 24 h) and picking individual colonies, which were used to inoculate batch cultures containing Gln− medium and incubated for 24 h (30°C). Amplification of the reporter gene was verified using flow cytometry (3).

For sequencing, cells were recovered from −80°C glycerol stocks (Fig. 1B) by plating the stocks and incubating them (YPD, 30°C, 24 h). For Illumina sequencing, strains were inoculated into rich medium (YPD) and grown overnight to a concentration of >1 × 10^8^ cells/mL (3). DNA was extracted using the Hoffman-Winston method (8), followed by a modified Nextera library preparation (9); the library was sequenced on a NextSeq 500 instrument in paired-end format (2 × 75 bp) (3). Adapters were trimmed using Cutadapt v1.12 with default settings (10). For Nanopore sequencing, strains were grown to a concentration of >1 × 10^7^ cells/mL in Gln− medium (11). DNA from each strain was extracted from ~1.5 × 10^9^ cells using the Qiagen Genomic-tip 20/G kit, following the manufacturer’s protocol. Nanopore was used for long-read sequencing following the manufacturer’s protocol (DGY1657, NBE_9065_v109_revB_23May2018; DGY1728 and DGY1744, NBE_9065_V109_revP_14Aug2019; all others, GDE_9108_v110_revJ_10Nov2020), with the following exceptions for DGY1657, DGY1728, and DGY1744: (i) incubation times for the enzymatic repair step were increased to 15 min, (ii) Agencourt AMPure XP beads were incubated for 30 min at 37°C before elution, and (iii) the adapter ligation time was increased to 10 min. All libraries were loaded onto FLO-MIN106D (R9.4) flow cells and sequenced using a MinION instrument (MIN-101B). Default parameters were used for all software unless otherwise specified. Demultiplexing of DGY1657, DGY1728, and DGY1744 was performed using Epi2Me (epi2me.nanoporetech.com). Base-calling was performed using Guppy v4.5.2, gpu (12). Long and short reads were used for hybrid assembly with MaSuRCA v4.0.9 (13). The assemblies were corrected with Ragtag v2.1.0 using the “remove-small” option to remove unique fragments shorter than 1,000 bp (14). LiftOff v1.6.3 was used for chromosome assignment, using the “copies” option to allow extra copies of genes (15). Proposed final assemblies of these genomes are shown in Fig. 1D to M.

### Data availability.

This whole-genome shotgun project has been deposited at GenBank under the accession numbers JAMQBG000000000 (DGY1657), JAMQBF000000000 (DGY1728), JAMQBE000000000 (DGY1734), JAMQBD000000000 (DGY1736), JAMQBC000000000 (DGY1740), JAMQBB000000000 (DGY1744), JAMQBA000000000 (DGY1747), and JAMQAZ000000000 (DGY1751). The versions described in this paper are the first versions, JAMQBG010000000 (DGY1657), JAMQBF010000000 (DGY1728), JAMQBE010000000 (DGY1734), JAMQBD010000000 (DGY1736), JAMQBC010000000 (DGY1740), JAMQBB010000000 (DGY1744), JAMQBA010000000 (DGY1747), and JAMQAZ010000000 (DGY1751). The raw Illumina reads are available under the SRA accession numbers SRR7057840 (DGY1657), SRR7057779 (DGY1728), SRR7057829 (DGY1734), SRR7057832 (DGY1736), SRR7057777 (DGY1740), SRR7057833 (DGY1744), SRR7057780 (DGY1747), and SRR7057828 (DGY1751). The ONT reads can be found under the SRA accession numbers SRR10525523 (DGY1657), SRR19440503 (DGY1728), SRR19440502 (DGY1734), SRR19440501 (DGY1736), SRR19440500 (DGY1740), SRR10525517 (DGY1744), SRR19440499 (DGY1747), and SRR19440498 (DGY1751).The sequencing data and accession numbers are provided in Table 1. The computational pipeline is available on GitHub (https://github.com/pspealman/WGS_assemble_pipeline [16]).

**TABLE 1:** Sequencing metrics for the S. cerevisiae strains in this study

Characteristic	Data for strain:							
	DGY1657	DGY1728	DGY1734	DGY1736	DGY1740	DGY1744	DGY1747	DGY1751
Illumina data								
SRA accession no.	SRR7057840	SRR7057779	SRR7057829	SRR7057832	SRR7057777	SRR7057833	SRR7057780	SRR7057828
Total no. of reads	3,653,321	6,229,610	1,760,584	5,401,867	4,531,223	5,447,512	4,709,699	3,272,815
Nanopore data								
SRA accession no.	SRR10525523	SRR19440503	SRR19440502	SRR19440501	SRR19440500	SRR10525517	SRR19440499	SRR19440498
Total no. of reads	47,436	194,350	44,825	40,245	88,706	140,456	30,289	343,924
*N*_50_ (bp)	8,324	625	1,929	1,954	1,198	414	2,449	2,074
Estimated coverage depth (×)	56.6	30.1	15.4	15.5	26.9	48.5	17.2	137.3
Assembly *N*_50_ (bp)	740,333	818,015	266,864	751,856	813,575	810,678	666,227	781,298
BUSCO (%)	96.10	99.30	90.20	98.60	99.40	99.40	98.90	97.50
GC (%)	38.20	38.10	38.30	38.20	38.30	38.30	38.30	38.20
No. of contigs	20	32	68	30	22	20	30	36
GenBank accession no.	JAMQBG000000000	JAMQBF000000000	JAMQBE000000000	JAMQBD000000000	JAMQBC000000000	JAMQBB000000000	JAMQBA000000000	JAMQAZ000000000
